# Physicians’ Perspectives on Prescription Alerts: A Journey Towards Reducing Fatigue

**DOI:** 10.7759/cureus.86996

**Published:** 2025-06-29

**Authors:** Makiko Takizawa, Noriyuki Nakayama, Yuko Ohishi, Kazumi Tanaka, Rei Noguchi, Yuichiro Saito, Keita Hirano, Yasuhiro Komatsu

**Affiliations:** 1 General Medical Center, Patient Safety, Saitama Medical University, Saitama, JPN; 2 Department of Healthcare Quality and Safety, Gunma University Graduate School of Medicine, Maebashi, JPN; 3 Department of Healthcare Quality and Safety, Gunma University Hospital, Maebashi, JPN; 4 Department of Pharmacy, Gunma University Hospital, Maebashi, JPN; 5 System Integration Center, Gunma University Hospital, Maebashi, JPN; 6 Department of Business Economics, Tokyo University of Science, Tokyo, JPN; 7 Department of Human Health Sciences, Kyoto University Graduate School of Medicine, Kyoto, JPN; 8 Department of General Medicine, Itabashi Chuo Medical Center, Tokyo, JPN

**Keywords:** alert fatigue, electronic medical records (emr), interrupted time series analysis, prescription alerts, quality improvement and patient safety

## Abstract

Background

Alerts in electronic medical records (EMR) and computerized decision support systems play crucial roles in enhancing patient safety by notifying healthcare providers of potential risks. However, an excessive number of alerts can lead to alert fatigue, in which clinicians become desensitized to notifications, potentially compromising patient safety. This study examined the experiences of physicians with alerts, focusing on their perceptions of and challenges posed by alert fatigue.

Methods

A mixed-methods approach was employed, combining an anonymous questionnaire survey with an interrupted time series (ITS) analysis. This study targeted physicians to gather insights into their experiences of using alerts. Data were collected through a Google Forms survey, and prescription data from the EMR were analyzed to assess the impact of the strategic reduction in non-critical alerts. Based on the survey feedback and research team discussions, the intervention targeted specific alerts contributing to alert fatigue, removing two types: “Powerful Drugs” and “Multiple Prescriptions.”

Results

While 76% of physicians found alerts helpful, 81% reported being overwhelmed by their volume, leading to alert fatigue. Notably, 55% of physicians admitted to dismissing alerts without reading them. The ITS analysis showed that a reduction of approximately 13,000 non-critical alerts per month did not significantly alter prescription behavior. The most problematic alerts were those related to administrative or cost issues, which contributed to the overall fatigue experienced by clinicians.

Conclusion

This study highlights the significant impact of alert fatigue on healthcare providers and underscores the need for a more streamlined alert system. Healthcare institutions can better manage alert fatigue and improve clinician efficiency and patient safety by focusing on reducing non-critical alerts and incorporating feedback from frontline clinicians.

## Introduction

Alerts are integral components of electronic medical records (EMR), and computerized decision support systems designed to enhance patient safety by notifying healthcare providers of potential issues and risks [1-4]. These alerts can cover a wide range of critical information, such as potential drug interactions, patient allergies, abnormal test results, and other clinical concerns that require immediate attention [5,6]. By flagging these issues, alerts aim to support clinicians in making informed decisions and preventing medical errors [7,8].

Although alerts are instrumental in enhancing patient safety, their excessive nature contributes to the significant challenge of alert fatigue [2,3,9-11]. Alert fatigue occurs when clinicians are overwhelmed by the sheer number of alerts. This issue is well-documented in the literature, including sources such as the European Commission against Racism and Intolerance (ECRI) white paper, which highlights the unintended consequences of excessive alerting [2,3,5,9-12]. As the number of notifications increases, healthcare providers may become desensitized to a constant stream of alerts, leading to reduced responsiveness to both important and less critical warnings [4,10,13-16].

Moreover, alert fatigue poses a significant threat to patient safety in the context of EMR and medication prescription errors [4,16]. The impetus for investigating alert fatigue arises from the critical role that hospital information systems play in preventing drug-related medical accidents [17]. Managing alert fatigue is paramount to improving patient safety by reducing the volume of notifications from computerized ordering systems in healthcare.

Multiple national reports, such as those from the Division of Adverse Event Prevention of the Japan Council for Quality Health Care (JQ) and the Medical Accident Investigation and Support Center of the Japan Medical Safety Research Organization, have addressed the urgent need to examine and mitigate alert fatigue [18]. At our academic institution serving patients in the Tokyo metropolitan area, the exploration of alert fatigue has gained momentum following certain instances. We observed cases suggestive of alert override leading to patient harm, prompting a detailed investigation into the phenomenon. To better understand this problem, we have searched the database from the Project to Collect Medical Near-Miss/Adverse Event Information of JQ using the keyword "medication x alert" for the period from 2019 to 2021. This process yielded 66 incidents and accident reports. Among these, 13 cases were identified in which the alert was in place but was bypassed or overridden, suggesting alert fatigue as a cause of adverse events [18].

Hence, this study focused on physicians' experiences with prescription alerts, aiming to uncover their perceptions and the challenges they face in managing the overwhelming number of alerts. We conducted an online survey and applied an interrupted time series (ITS) analysis to address alert fatigue and to support quality and safety officers in acting as boundary spanners and coordinators within their organizations. The objectives of this study were to examine physicians’ experiences and perceptions related to alert fatigue from prescription alerts and to evaluate the impact of reducing non-critical alerts on prescribing behavior and clinical workflow.

## Materials and methods

Study design

We employed a mixed-methods approach combining an anonymous questionnaire survey and ITS analysis.

**Figure 1:**
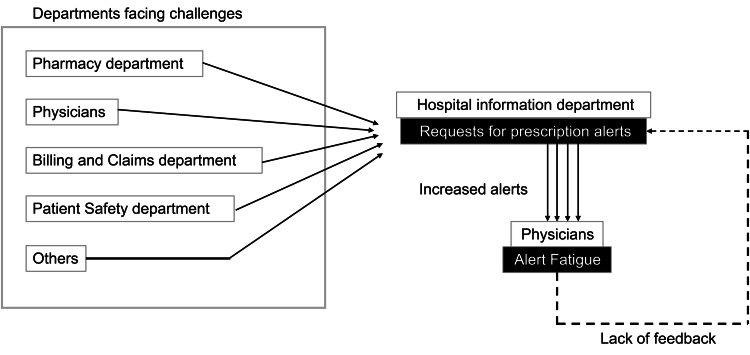
Flowchart of challenges leading to prescription alert fatigue This flowchart illustrates the sequence of challenges faced by various departments in a hospital, functioning as silos, that lead to increased prescription alerts. These alerts, processed by the hospital information department, contribute to alert fatigue among physicians, creating a feedback loop of challenges and alert fatigue. This figure was created by the authors.

This design was selected for a comprehensive exploration of alert fatigue among healthcare practitioners and its implications for patient safety. As illustrated in Figure 1, this issue is considered a systemic challenge stemming in part from the lack of feedback from physicians and the fact that, although each department strives within its own professional silo, coordinated efforts across the system are lacking. Based on the survey findings and expert discussion, specific prescription alerts contributing to alert fatigue were identified and discontinued as an intervention. The ITS analysis quantitatively evaluated changes in prescribing behavior before and after the alert removal.

Participants in the survey

A total of 615 physicians and dentists from various clinical departments, who were employed at a university hospital during the study period, were included in this study. The participants aimed to capture insights from healthcare professionals who were directly involved in the prescription process. All the physicians and dentists employed at the hospital during the study period were contacted for voluntary participation. The inclusion criteria encompassed all physicians and dentists who willingly participated in the survey and provided informed consent. Incomplete responses were excluded from the analysis.

Data collection instruments

Anonymous Questionnaire Survey

To capture healthcare practitioners' perceptions of prescription alerts, concerns, and attitudes toward alert fatigue, Google Forms was used to conduct an anonymous questionnaire survey. All physicians in the various departments completed the survey, and the inclusion criteria were based on voluntary responses with complete consent. The questionnaire collected data on several variables, including the frequency with which physicians prescribed medication, the duration of their clinical practice (categorized into ranges such as 1-5 years, 6-10 years, etc.), and their specific departments.

To assess the impact of the intervention on prescription behavior and identify trends in alert responses over time, an ITS analysis was performed. EMR, specifically prescription data, served as the primary data source for the ITS analysis. This study implemented a strategic reduction in noncritical alerts based on collaborative efforts and feedback from healthcare practitioners. The Google Forms survey link was distributed to clinical department secretaries to ensure broad participation. Anonymity was maintained throughout the collection. The survey was conducted over a 20-day period from February 9 to February 28, 2022. To improve the response rate, reminders were sent twice during the period, and responses were collected voluntarily.

Data Collection for ITS Analysis

Prescription data were extracted from the hospital EMR. The number of monthly prescriptions that met the predefined conditions for alert generation was counted. The ITS analysis involved tracking prescription patterns before and after the intervention to evaluate their impact. The initial phase included 12 time points before and after the intervention. As outcome variables of ITS analysis, the prescription rate of powerful drugs was calculated by dividing the number of prescriptions of powerful drugs by the total number of prescriptions each month, whereas to calculate that of seven or more drugs, the number of prescriptions for which there were seven or more drugs was divided by the total number of prescriptions each month.

Ethical considerations

The survey ensured participants' anonymity and voluntary participation. Respondents were explicitly informed that completing the questionnaire would be considered as providing informed consent. Prescription data were anonymized to ensure confidentiality. The study was approved by the Ethics Committee for Medical Research Involving Human Subjects at Gunma University (Approval No. HS2020-188), and institutional ethical guidelines were adhered to throughout the study.

Statistical analysis

Descriptive statistics were used to summarize participants' perceptions, concerns, and attitudes.

The open-ended responses were qualitatively analyzed to identify common themes. The ITS statistician conducted an analysis to assess changes in prescription behavior following the intervention. ITS analysis is a valuable analytical method for evaluating the effectiveness of health interventions or health policies and is widely used for studies from clinical intervention to public policy [19,20]. In ITS model development, autoregressive, moving average, and autoregressive moving models were tested to consider the effects of autocorrelation, and a model with the lowest Akaike’s information criteria (AIC) was selected for each intervention study. Trends and statistical significance were examined to determine the effectiveness of the interventions using each model. All analyses were performed using R version 4.4.2 (The R Foundation for Statistical Computing, Vienna, Austria).

## Results

Responses were obtained from 154 participants, yielding a response rate of 25.0%. Respondents represented various clinical departments and levels of experience. Respondents included pediatrics, gastroenterology, urology, and other specialties (Table 1) with the following distribution: 28 physicians with 1-5 years of practice, 22 with 6-10 years, 31 with 11-15 years, 26 with 16-20 years, 31 with 21-25 years, 15 with over 26 years of experience, and one respondent who did not disclose their experience level. Table 2 shows how frequently the participants prescribe medications in actual clinical practice.

**Table 1:** Distribution of survey participants by clinical departments and years of practice Departments were grouped into four categories: Internal Medicine, Major Surgery, Minor Surgery, and Others. The classification details are provided below. Clinical experience indicates the total number of years since graduation. *1 Internal medicine: Pediatrics, Gastroenterology/Hepatology, Endocrine Diabetes Internal Medicine, Nephrology/Rheumatology, Hematology, Respiratory/Allergy Medicine, Neurology, Cardiology, General Medical Department *2 Major surgery: Gastrointestinal surgery, hepatobiliary and pancreatic surgery, respiratory surgery, and neurosurgery *3 Minor surgery: Urology, Obstetrics and gynecology, Orthopedic surgery, Dermatology, Dental oral and maxillofacial surgery, Otorhinolaryngology, Ophthalmology, Breast gland/endocrine surgery, Pediatric surgery, Plastic surgery *4 others: Emergency Department, Anesthesiology and Resuscitation Department, Clinical Training Center, Clinical Laboratory Department (Laboratory Department), Rehabilitation Department, Department of Nuclear Medicine, Infection Control Department, Prefer not to answer. Psychiatry and Neurology, Radiology, Do not answer.

Department	1st-5th Year	6th-10th Year	11th-15th Year	16th-20th Year	21st-25th Year	26+ Years	No Answer	Grand Total
Internal medicine*1	5	6	7	11	16	10	0	55
Major surgery*2	2	3	6	4	1	1	0	17
Minor surgery*3	9	8	8	7	10	1	0	43
Others*4	12	5	10	4	4	3	1	39
Total number of participants	28	22	31	26	31	15	1	154

**Table 2:** Self-reported frequency of prescribing medications in clinical practice Participants were asked how often they prescribe medications to real patients in their routine clinical practice. Response options reflect frequency categories, and the total number of respondents was 154.

Frequency	Count
4 or more days a week	69
About 1-3 days a week	66
About 1-3 days a month	12
Do not prescribe	4
A few days a year	3
Total	154

Four of the 154 respondents do not prescribe and were excluded from the prescription-alert perception questions; Table 3 therefore reflects the responses of the remaining 150.

**Table 3:** Survey results on physicians’ perceptions and behaviors regarding prescription alerts This table presents survey results on physicians’ views regarding prescription alerts, including perceived usefulness, concerns about excessive volume, and behaviors such as ignoring or dismissing alerts. Responses reflect the dual role of alerts in supporting patient safety and contributing to cognitive burden.

Question	Yes/Strongly agree	Agree	Disagree	No/Strongly Disagree
Have you ever prevented critical patient safety events due to prescription error by prescribing alerts?	59 (39%)	NA	NA	91 (61%)
The alerts displayed at the time of prescription are useful for medical practice.	16 (10%)	99 (66%)	28 (19%)	7 (5%)
The number of alerts displayed at the time of prescription is excessive.	18 (12%)	104 (69%)	27 (18%)	1 (1%)
I feel like I am losing attention to individual alerts.	18 (12%)	84 (56%)	46 (31%)	2 (1%)
I sometimes dismiss individual alerts without examining the content of the alerts.	23 (15%)	60 (40%)	54 (36%)	13 (9%)
I feel like I might miss out on important patient safety alerts in my day-to-day practice.	9 (6%)	93 (62%)	46 (31%)	2 (1%)
The number and content of alerts displayed during prescription are appropriate for me.	8 (5%)	69 (46%)	61 (41%)	12 (8%)

The survey revealed complex perceptions of prescription alerts among physicians. While 76% of the respondents found prescription alerts to be generally helpful, with 39% acknowledging that these alerts had previously helped prevent medication errors related to patient safety, there was also significant concern about the volume of alerts. Notably, 68% of the physicians expressed worry about missing important patient safety alerts because of the overwhelming number they receive.

Alert fatigue was evident, with 81% of respondents reporting that the number of alerts they receive is excessive. This overwhelming volume contributed to cognitive overload, leading 68% of the physicians to admit that they feel less focused on alerts. The high number of alerts and their low specificity have raised concerns among physicians. More than half the respondents (55%) reported that they sometimes dismissed alerts without thoroughly reading them, which increased the risk of overlooking important safety information (Table 3).

The survey also identified the most problematic alerts from physicians’ perspectives. The "Passed Prescription Deadline" alert, which indicates that pharmacists would not check prescriptions after a certain time, was ranked as the most problematic. Following this, the "Prescriptions with Seven or More Drugs" alert, which notifies physicians that the hospital’s reimbursement will be reduced for prescriptions containing more than seven drugs, an initiative by the government aimed at reducing polypharmacy, and asks if the prescription could be changed, was the second most disliked. Lastly, the "Powerful Drug" alert, which merely informs the physician without requiring immediate action, was ranked third. These alerts, particularly those related to administrative or cost issues, are considered less relevant to patient safety and contribute to the overall problem of alert fatigue. The survey included 35 types of prescription alerts commonly encountered in the hospital’s EMR system. While it is not possible to fully identify all alert types implemented in the system, the survey focused on those most relevant to physicians' routine clinical work.

**Figure 2:**
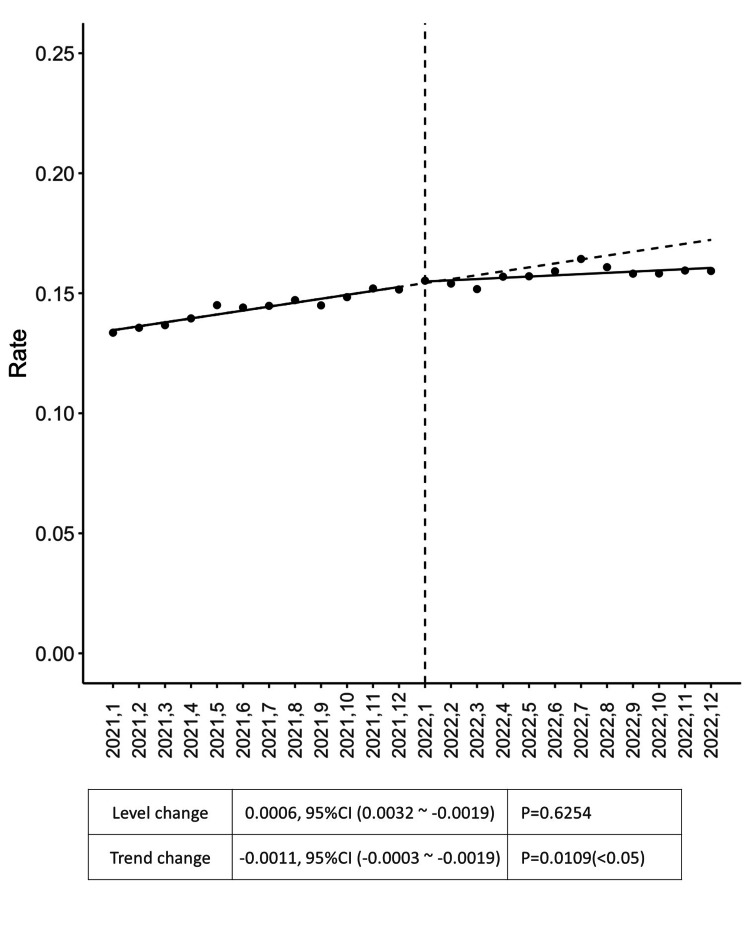
Prescription rate of powerful drug Statistical analysis uses interrupted time series analysis to show the pre- and post-intervention results. In the scatter plot, the X-axis represents time, and the Y-axis indicates the rate. The line graph illustrates the prescription rate of a powerful drug over time, from January 2021 to December 2022. The vertical dashed line at "2022.1" indicates the intervention. Both the level and the trend changes are shown, and the trend change was statistically significantly reduced after the intervention (p<0.05).

In response to these findings, the Patient Safety and Medical Information departments reviewed and decided to remove alerts that were not critical to patient safety and posed minimal risk if omitted. Two types of alerts were removed, namely “Powerful Drugs” and “Multiple Prescriptions.” Although difficult, this decision was guided by the need to reduce the overwhelming number of alerts and mitigate the risk of alert fatigue. Both prescription alerts were interruptive (warning-type) alerts, which allowed physicians to proceed with prescribing after deleting the alert comments. The effect of removing a “Powerful Drug” alert on prescription behavior was analyzed using an ITS approach. The graph shows a significant trend decrease in the prescription rate of powerful drugs following the removal of the alert, although the level remained unchanged (Figure 2). The vertical axis represents the prescription rate, and the horizontal axis denotes time. Statistical data highlights a level change of 0.0006, p=0.6254, and a trend change of -0.0011, p=0.0109.

**Figure 3:**
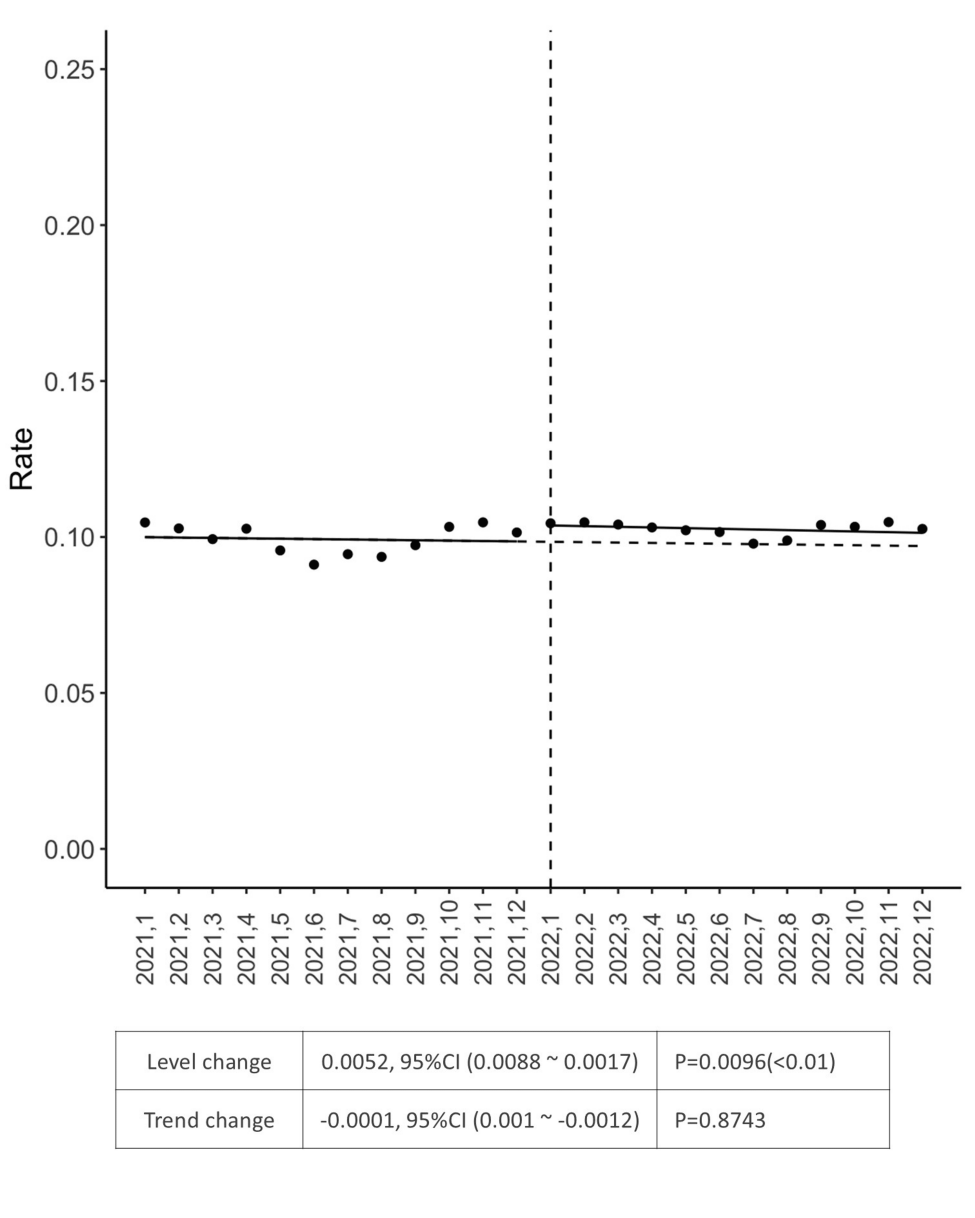
Prescription rate for seven or more drugs The statistical analysis uses interrupted time series to show the pre- and post-intervention results. In the scatter plot, the X-axis represents time, and the Y-axis indicates the rate. This line graph illustrates the prescription rate for seven or more drugs over time, from January 2021 to December 2022. The vertical dashed line at "2022.1" indicates the intervention. Both the level and the trend changes are shown, with a statistically significant increase in level after the intervention (p<0.05).

Further temporal analysis of data on prescription rates for seven or more drugs revealed a significant level increase over time (Figure 3). The statistical analysis yielded a level change of 0.0052 with a p-value of 0.0096 and a trend change of -0.0001 with a p-value of 0.8743.

Based on prescription data, an average of approximately 2,500 prescriptions per month include seven or more drugs, and around 10,500 involve powerful drugs, both of which trigger frequent alerts. Interrupting these two types of alerts is expected to reduce cognitive overload among physicians by eliminating an average of approximately 13,000 alert interruptions per month. Although a slight increase was noted in prescriptions involving seven or more drugs, no patient harm events were observed in association with this intervention.

## Discussion

This study offers valuable insight into the complex issue of alert fatigue in healthcare settings, particularly in the context of prescription alerts. These results highlight both the critical role that alerts play in patient safety and the unintended consequences of alert overload, which can paradoxically undermine the safety that they are designed to protect. Our survey revealed that, while the majority of physicians recognize the importance of prescription alerts, there are significant concerns about the number and specificity of these alerts. A significant number of respondents expressed worry about missing important patient safety alerts owing to alert fatigue, and over half admitted to occasionally deleting alerts without reading them thoroughly. These findings underscore the dual-edged nature of alerts; although some are essential for preventing medication errors, the frequent appearance of low-priority or administratively driven alerts may diminish clinicians’ ability to distinguish truly safety-critical messages.

Alert fatigue stems from a combination of factors, including the high volume of alerts, the low specificity of many of these alerts, and the repetitive nature of certain notifications [3,6,10,15,21,22]. The observations made by these authors, ranging from single-institution studies to randomized trials in highly specialized care, were similar [3,6,10,15,21]. The results of our survey also indicate that physicians often feel overwhelmed by the sheer number of alerts, many of which they perceive as irrelevant or unhelpful. This overload, as observed in multiple studies, leads to desensitization in which critical alerts are missed or dismissed, thereby increasing the risk of medication errors [1,3,4,13,17,21]. These observations are in line with our findings in which over half of the physicians admitted to deleting alerts without reading them.

Significant effort has been devoted to understanding this burden [3,7,10,11,14,21]. One of the key issues identified in this study was the lack of feedback mechanisms for physicians regarding the alerts they received [1,17]. The open-ended responses from the present survey indicated a strong desire among physicians for regular surveys and feedback opportunities to ensure that the alerts are relevant and useful. The absence of such a mechanism represents a significant design flaw in the current system, contributing to the proliferation of unnecessary alerts and the resultant alert fatigue. A systematic review of alert optimization has suggested that committee involvement, clinician feedback, and the use of alert data are important elements for improving alert systems [23]. To address these challenges, we propose a system redesign approach that includes the establishment of an alert management team and a feedback loop involving stakeholders. This team functions as a boundary spanner, bridging the gap between frontline physicians and technical systems that generate alerts. The alert management team is composed of physicians and pharmacists from the patient safety department, along with staff from the information systems division. This multidisciplinary team aims to support appropriate alert use and reduce alert fatigue by monitoring the overall volume of prescription alert types to prevent excessive increases, gathering feedback from physicians, and making decisions on which alerts to modify, reduce, or eliminate. This approach aligns with recommendations from the ECRI, which advocate organizational awareness of alert fatigue and the need to control the overall number of alerts [5].

The study also explored specific alert types identified as particularly problematic, such as alerts for powerful drugs and those related to prescriptions of seven or more drugs. The ITS analysis performed for these two alerts revealed that the successful alleviation of alert load did not adversely alter prescription practices in terms of patient safety. There was a significant increase in the prescription rate of seven or more drugs, although the trend has not changed. This increase may be partly attributed to the effect of the intervention; however, it should also be interpreted in light of a possible confounding factor, the impact of the COVID-19 pandemic. The region was severely affected between May and June of 2021, and a declaration of emergency was announced in August and September of 2021. These events correspond to dips in Figure 3, which may have affected the data. The alert management team recommended shifting the guidance regarding prescriptions involving seven or more drugs from real-time alerts at the point of prescribing to periodic notifications through management reports. While the trend for the powerful drug alert has shifted, it could be affected by the increase in the number of types of potent drugs that can be prescribed. University hospitals are implementing the latest pharmacological treatments, and in fact, during the year 2021, the number of types of potent drugs prescribed increased (data not shown). On the other hand, in 2022, the number of types of potent drugs prescribed remained flat. It should also be noted that this period coincided with the declaration of a state of emergency due to the COVID-19 pandemic, which significantly affected healthcare providers' behaviors.

With respect to the optimization of alerts in EMR systems, McGreevey et al. provided a comprehensive review of governance and optimization strategies for EMR alerts, focusing on broader institutional approaches to managing alert burdens [21]. They emphasized the need for a balanced approach to alert management that improves patient care while reducing the clinician burden, which is consistent with our findings. Furthermore, our study extends this understanding by documenting the direct impact of reducing specific alerts on prescription behavior (Figures 2, 3), thus providing a more granular view of how alert management decisions influence clinical practice. 

Considering the issue of overriding alerts, Topaz et al. evaluated drug allergy alerts and their overriding rates, revealing a high override rate of 86.3%, which increased over time [16]. The alerts for drug allergies, particularly those associated with narcotics and statins, are frequently overridden, often because alerts are triggered by non-life-threatening reactions or probable matches rather than definite allergies [16]. Similar observations were highlighted in a review by Poly et al., in which drug-drug interactions, old age-related alerts, and renal alerts were classified as inappropriate [13]. Understanding the nature of alerts and weighing their impact on patient care is paramount [6]. This has been evaluated in our study by the observation of ITS analysis as part of the alert management team’s intervention on the powerful drug alert and polypharmacy.

A few authors have leveraged machine learning capabilities [24,25], and the accumulated data points to train these systems are no longer a highly valuable resource available only at certain institutions. With the widespread use of EMR and alert systems, machine learning strategies have targeted irrelevant alerts and reduced the alert volume by more than half (54.1%) [24,25]. When implemented collectively with upcoming and existing technologies, these strategies enhance patient safety.

**Figure 4:**
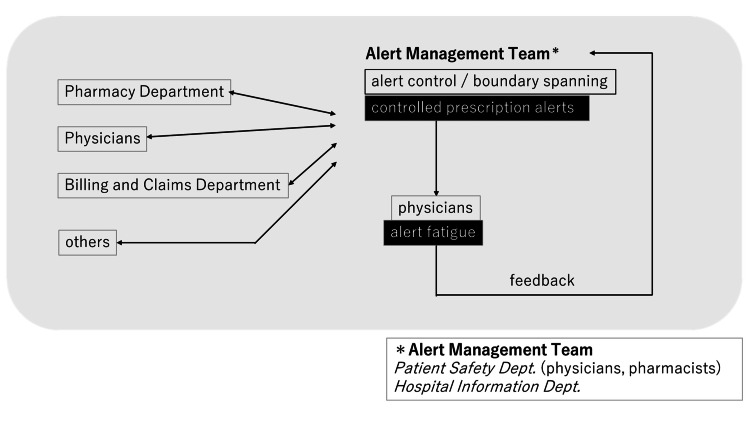
Alert management team and controlled prescription alerts workflow The alert management team, composed of members from the Patient Safety Department (physicians, pharmacists) and the Hospital Information Department, coordinates institution-wide prescription alert management by working with departments such as Pharmacy, Clinical Divisions, and Billing/Claims. The team aims to reduce alert fatigue and support system improvement through ongoing collaboration. This figure was created by the authors.

To address these challenges, we propose a system redesign with an alert management team that bridges the gap between physicians and technical systems. This team monitors alerts, gathers feedback, and makes data-driven decisions to improve the system. Figure 4 illustrates the workflow of the alert management team, in which a multidisciplinary group from the Hospital Information Department and Patient Safety Department collaborates with multiple departments, such as Pharmacy, Physicians, and the Billing and Claims Department, to manage alert systems at the institutional level.

Our study has certain limitations. The most significant factor is the confinement of the study to a single institution, which may limit the generalizability of our findings. Nevertheless, the methodology employed, using a questionnaire survey and collaborative efforts to improve alert systems, has the potential to be applied more broadly across different healthcare settings. Existing literature on alert fatigue often focuses on describing problems or discussing structural issues; however, few studies have implemented and evaluated interventions as this one did.

Another limitation is the absence of quantitative event data related to alerts, such as the frequency and types of alerts issued to specific physicians and whether these alerts were accurate. This limitation is due to the fact that our EMR system lacks the functionality to track the number and content of alerts or assess their influence on prescribing behavior. Therefore, we used prescription counts as a substitute measure, as no direct tracking of alert data was available, although this limits our ability to evaluate behavioral changes directly. Future EMRs should enable alert tracking and incorporate AI-based analytics to better understand and address alert fatigue. Such data would have provided a more granular understanding of the alert landscape and specific triggers of alert fatigue.

Furthermore, the study period overlapped with the COVID-19 pandemic, including a regional state of emergency. These events may have influenced both prescribing behavior and physicians’ alert responsiveness. Although temporal fluctuations were observed, particularly during pandemic waves, the magnitude of this confounding effect remains uncertain and could not be quantitatively adjusted in this study.

Despite these limitations, the key findings aligned with the strategies recommended in the literature, such as those outlined in the ECRI white paper. The focus should be on fostering constructive discussions with vendors regarding the alert design to ensure that clinical decision systems are effective and sustainable. Vendors should be strongly encouraged to provide functionalities that enable institutions to systematically monitor alert frequency, override behavior, and alert outcomes. In the future, the incorporation of AI technologies that support adaptive, user-specific alerting may also offer promising avenues for minimizing alert fatigue while maintaining clinical relevance. Collaborative efforts across departments, systems, and vendors are essential for maximizing the usefulness of current technologies while minimizing the burden on clinicians. We have demonstrated that the volume of alerts can be reduced without compromising patient safety, paving the way for further innovations in alert system design and implementation.

## Conclusions

This study highlights the challenge of balancing the benefits of prescription alerts with the risks of alert fatigue in healthcare settings. Although alerts are crucial for patient safety, their excessive number and low specificity can overwhelm physicians, causing critical alerts to be overlooked. Our findings suggest that targeted reductions in unnecessary alerts guided by frontline feedback and managed by a dedicated alert management team can help mitigate alert fatigue and enhance the effectiveness of clinical decision support systems. This approach offers a pathway for improving both physician workflow and patient safety outcomes.
